# The Impact of Different Drying Techniques on the Physicochemical and Quality Characteristics of Oil Palm Fruit Mesocarp (*Elaeis guineensis*)

**DOI:** 10.1155/2021/2005502

**Published:** 2021-12-24

**Authors:** Vida Gyimah Boadu, Edward Ken Essuman, Gifty Serwaa Otoo, Kate Bigson

**Affiliations:** ^1^Department of Hospitality and Tourism Education, Akenten Appiah-Menka University of Skills Training and Entrepreneurial Development, Kumasi, Ghana; ^2^Department of Nutrition and Dietetics, School of Allied Health Sciences, University of Health and Allied Sciences, Ho, Ghana; ^3^Department of Food Science and Postharvest Technology, Cape Coast Technical University, Cape Coast, Ghana; ^4^Department of Hotel, Catering and Institutional Management, Dr. Hilla Limann Technical University, P.O. Box 553, Wa, Ghana

## Abstract

Drying is one of the traditional means of preserving food. However, various drying methods can influence the nutritional and bioactive constituents of the food product. This study is aimed at evaluating the effect of different drying methods on the proximate composition, physicochemical properties, and minor constituents of palm oil fruit mesocarp. Two varieties of fresh oil palm fruit (dura and tenera) were processed to separate the mesocarp from the other part of the fruit. The fresh fruit mesocarp was divided into five groups and subjected to different drying methods. Proximate and physicochemical characteristics of the oil palm fruit mesocarp were determined. The dried mesocarp had low moisture content (1.49-3.28%), high crude fat content (78.10-90.60%), carbohydrate (4.41-15.12%), crude protein (0.93-3.40%), and ash (0.53-1.15%). The free fatty acid (FFA) (1.06-3.54) and acid value (AV) (2.17 to 8.83 mgKOH/g) were lower because the samples were heated at 100°C for 30 min. The lower pH, FFA, AV, titratable acidity, moisture content, and high antioxidant activity of the oil palm fruit products could be an indication of shelf stability against microbial contamination and rancidity.

## 1. Introduction

The oil palm (*Elaeis guineensis*), commonly called the African oil palm, is a monocotyledon belonging to the genus *Elaeis*. It is a perennial plant and has the highest production per hectare among oil crops, yielding an average of 3.7 tons of oil per hectare per year [1]. Oil palm fruit is native to the West African region particularly Côte d'Ivoire, Ghana, Nigeria, and Sierra Leone. Dura and tenera are the two main varieties cultivated in Ghana [2]. The oil palm fruit is a major source of oil for human food use and secondary industrial uses. It is an important part of the local nutrition customs and a significant product of global commercial importance. It has been widely introduced throughout the tropics and has recently seen a surge in plantation establishment due to the increased interest in biofuels. It has the reputation of “the world oil king.”

Two types of oil can be extracted from the oil palm fruit, red palm oil from the fleshy mesocarp and palm kernel oil from the palm kernel [3]. Palm fruit also contains phytonutrients that can provide the oil with nutritional and health beneficial properties. These phytonutrients include vitamin E, carotenoids, squalene, sterols, phospholipids, and glycolipids [4]. The quality of palm oil extracted from the fruit is determined by different factors, and free fatty acid (FFA) is one of the most frequently used quality indicators during production, storage, and marketing. Other parameters that dictate the price of a palm oil product include moisture, impurities, and peroxide value (PV). These parameters could also be of major importance in checking the quality of the oil palm mesocarp.

Ripe palm fruits are easily attacked by fungi and do not have appreciably shelf life. The average shelf life of the ripe fruit is three days. Gamma radiation has been reported to extend the shelf life of ripe oil palm fruit mesocarps [5]. However, drying is an ancient process used to preserve and prolong the shelf life of various food products [6]. Although drying affects the nutritional composition and bioactive compounds of fruits [7, 8], the processing and storage methods are equally significant in the preservation of useful components for health. This can be achieved through the appropriate selection of drying temperature, time, and methods. Oven, solar, and freeze-drying have been successfully employed to produce flour with sufficient nutrient composition and quality from African Palmyra fruit [9]. The main aim of drying food products is to eliminate water in the solid to a level at which microbial spoilage and deterioration resulting from chemical reactions are significantly reduced [10]. This enables the product to be stored for longer periods since the activity of microorganisms and enzymes is inhibited through drying.

The proper and fruitful integration of oil palm fruit into food systems depends on its physicochemical and nutritional composition. Currently, there are few reports on the nutritional composition and physicochemical properties of the oil palm mesocarp and most of the data are from Indonesia, China, and Malaysia [11]. There are almost no data about the effects of drying techniques to preserve the nutritional constituents of the oil palm fruit mesocarp. This study is aimed at evaluating the effect of different drying methods on the proximate composition and physicochemical properties and minor constituents of palm oil fruit mesocarp.

## 2. Materials and Methods

### 2.1. Sources of Materials

Fresh and matured oil palm fruits (*Elaeis guineensis*) were purchased from Bantama market, Kumasi, Ghana. These include two varieties of oil palm fruit (dura and tenera), and each variety was treated as one sample. All chemicals and equipment used in the analysis were of analytical grade and were obtained from the Food Science Laboratory of the Department of Food Science and Technology, Kwame Nkrumah University of Science and Technology, Ghana.

### 2.2. Sample Preparation

**Figure 1:**
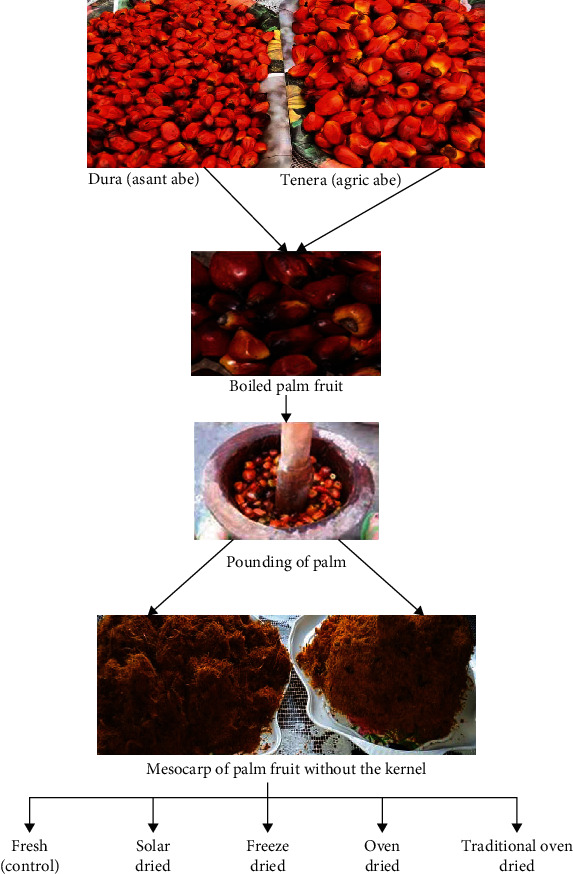
Flowchart for obtaining mesocarp from palm fruit for different drying techniques.

The two varieties of oil palm fruits were separately washed and weighed after removing foreign materials. The oil palm fruits were pared to break the flinty shell. They were boiled for about 30 min at 100°C, strained off the water, and pounded in a mortar to free the mesocarp from the nuts. The nuts were then removed by hand, and the mesocarp was allowed to attain room temperature. They were divided into five portions and weighed using an electronic balance. A portion was solar dried for 72 h using the solar drier. The second portion was freeze-dried (at 47°C to -55°C, 0.002 to 2.7 Torr) for 72 h using a vacuum freeze dryer (Model: YK-118-50 Taiwan); the third and fourth samples were dried at 60°C for 6 h using the hot air oven (Binder Heating and Drying Oven, Tuttlingen, Germany) and a traditional or clay oven at a temperature between 50°C and 60°C, respectively. The fifth portion was used as the experimental control in the analysis to be carried out. The samples were packaged in ziplock bags and stored at -20°C in a freezer for further analysis. The process of sample preparation is shown in Figure 1. In all, a total sample of ten (10) from two varieties of palm fruits were used for this study and each experiment was repeated three times and the average recorded.

### 2.3. Proximate Analysis of the Palm Mesocarp

Proximate analysis was done on both fresh and dried palm fruit samples. Moisture, protein, ash, crude fat, and crude fibre were determined according to AOAC [12]. Protein was calculated from total nitrogen using the conversion factor of 6.25. Carbohydrate was determined by difference. All experiment was carried out in triplicate.

### 2.4. Titratable Acidity

Five grams (5 g) of each sample was weighed into a 200 mL conical flask, and 50 mL of distilled water was added. The procedure by Nielsen [13] was followed to determine titratable acidity.

### 2.5. Acid Value (Acid Number) and Free Fatty Acid

Sufficient oil (about 50 mL) was extracted from each of the five samples and placed in a dried conical flask, and the method for acid value and free fatty acid by AOAC [12] was followed for the determination. The percentage of free fatty acid was calculated by multiplying the acid value by 0.503. All experiments were carried out in triplicate.

### 2.6. Antioxidant Activity Determination

For each of the samples, 0.5 g was weighed into a 15 mL centrifuge tube containing 10 mL of distilled water and centrifuged at 10,000 rpm for 15 min. For each extract obtained, 0.2 mL distilled water and 6 mL of 0.004% DPPH (1,1-diphenyl-2-picrylhydrazyl) were put into a 6.4 mL reaction mixture container and shaken by hand. It was kept in the dark for 30 min at room temperature. The absorbance of the reaction mixture and blank was read at 517 nm. The ability to scavenge the DDPH was calculated as

(1) $$\begin{array}{c} \text{DPPH radical scavenging activity }\left(\%\text{of Inhibition}\right)=\left[1-\left(\frac{A_{\text{S}}}{A_{0}}\right)\right]\times 100, \end{array}$$

where *A*_S_ is the absorbance of the sample and *A*_0_ is the absorbance of DPPH solution diluted with the same volume of distilled water.

Distilled water was used as blank.

### 2.7. Carotenoid Estimation

The estimation of carotenoid contents of the samples was determined according to the method described by Mackinney [14] and Maclachlan and Zalik [15], respectively.

### 2.8. Peroxide Value

For each sample, 0.5 g was weighed into a 250 mL glass-stoppered Erlenmeyer flask and 30 mL of the acetic acid-chloroform solution (480 mL acetic acid and 320 mL chloroform) was added. The flask was swirled until the sample was completely dissolved by carefully warming on a hot plate. A pipette was used to add 0.5 mL of saturated potassium iodide solution to the content and was swirled for one minute. Distilled water (30 mL) was added and shaken vigorously to liberate the iodine from the chloroform layer. A burette was filled with 0.1 N sodium thiosulfate, and 1 mL of starch solution was used as an indicator. Titration was performed, and the volume of titrant used was recorded.

### 2.9. Statistical Analysis

The means and standard deviation of all three replications were analysed using the Statistical Package for Social Sciences (SPSS, IBM SPSS Statistics v20). The differences among the test parameters were identified by one-way analysis of variance (ANOVA) using Tukey's test. All statistical tests were carried out at a 5% significance level.

## 3. Results and Discussion

### 3.1. Proximate Composition of Oil Palm Fruit

The moisture content of the fresh pulp palm fruit was high with values of 26.36% and 31.15% for the two varieties. The moisture content of the dried palm fruit samples ranged from 1.49 to 3.28, with freeze-dried samples from the two varieties recording the highest moisture while the solar-dried sample recorded the lowest (Table 1). The moisture content of all samples was higher than the values 0.14-0.16% and 0.411-0.175% as reported by Udensi and Iroegbu [16] and Ali et al. [17] for palm oil, respectively. Food products with high moisture content are prone to microbial attack and spoilage and consequently have limited shelf life [18]. Therefore, the low moisture content of the dried samples shows their stability against microbial contamination and longer shelf life.

The ash content of the dried palm fruit ranged from 0.53% (freeze-dried) to 0.79% (solar-dried fruit) (Table 1). All dried fruit samples and the fresh pulp had slightly lower ash than the value (1.11%) reported in the literature for the oil palm fruit obtained from China [11]. The crude fat concentration ranged from 78.10% (freeze-dried palm fruit) to 90.03 (solar-dried palm fruit). This is an indication that fat is a major constituent of palm fruit and a good source of oil [11]. The crude fat content recorded for the fresh pulp was low due to the high moisture for both varieties (26.36% and 31.15%) as reported for fresh African Palmyra palm fruit [9]. The polar nature of water might have affected the solvent extraction procedure. The crude protein content of the dried sample ranged from 0.93 to 2.40% (Table 1), with the freeze-dried sample recording the least value (0.93%) while the electrical oven-dried sample recorded the highest protein content (3.40%). However, the majority of the fruit dried samples had higher protein content than the fresh pulp (2.03%) as reported in previous studies [11]. The freeze-dried samples of the tenera variety recorded the highest value (15.12%) of carbohydrate while the dura samples recorded the least (4.41%). Dura comprises mesocarp content of 35-55% but tenera has medium to high mesocarp content of 60-95% by weight of the fruit [19]. This is because tenera contains a lot of pulp (with a thick mesocarp).

### 3.2. Physicochemical Properties of Oil Palm Fruit Mesocarp under Different Drying Methods

The acid value (AV) of the samples ranged from 2.17 to 8.83 mgKOH/g (Table 2). The values recorded for the samples were lower than the value (51.65 mgKOH/g) reported in the literature [11]. The major determinant of Africa palm fruit and oil is free fatty acid (FFA). A measure of total acidity of lipids related to contributions from all constituents' free fatty acids is the acid value. The outcome of oxidation of FFA is rancidity, and this affects the oil quality of the fruit.

The FFA of the samples ranged from 1.10 to 4.44% and was lower than the value reported in the literature (24.30%) [11]. The values were also lower than the maximum concentration value of 5.0% for crude palm oil, Codex 210 [20]. The results show a low level of FFA for all drying methods for the two varieties. However, both varieties (tenera and dura) of solar drying recorded the highest values of 3.55 and 4.44%, respectively. AV and FFA observed in this study were very low compared to those reported by Li et al. [11]. The difference could be due to the boiling of the palm fruit at 100°C for 30 min before drying as observed in this study. The low levels of FFA in the samples indicate that the products may be stored for a longer period without spoilage through oxidative rancidity [21].

The pH of the palm oil fruit ranged from 4.61 to 5.73 as shown in Table 2. The solar-dried products had the lowest pH while the freeze-dried product has the highest value. The pH is a measure of the concentration of free hydrogen in a solution. It helps in assessing the ability of microorganism growth in food [22]. The titratable acidity of the palm oil fruit ranged from 0.35 to 1.06 (Table 2). Titratable acidity is a measure of the total acid concentration in food. It is a better predictor of how organic acids influence flavour, colour, microbial stability, and good keeping quality in food. However, inorganic acids play a significant and major role in food acidulation [23]. The lower pH, titratable acidity, and moisture content of the oil palm fruit mesocarp could be an indication of shelf stability against microbial growth [24].

Peroxide values (PV) of the variously treated mesocarps ranged from 1.92 to 17.81 meq/kg (Table 2). The solar-dried products had the highest value while the freeze-dried product recorded the lowest value. The peroxide value for both varieties of the oil palm fruit mesocarp of the solar-dried products was higher than the values recorded in the literature (3.22 meq/kg) [11]. PV in palm oil points out the state of oxidation of a substance. If oxidation continues for long, it makes the oil rancid and gives an unpleasant smell to the substance. This phenomenon is influenced by the temperature of preservation and by the interaction with air and light. PV of oil palm fruit must not exceed the upper limit (15 meq/kg) as established by Codex 210 [20]. The high PV value for solar samples was due to exposure to light during drying [25]. The discovery of peroxide gives evidence of rancidity in unsaturated fats and oils in foods. A high PV value may increase the formation of hydroperoxide or either increase decomposition.

### 3.3. Beta-Carotene and Antioxidant Properties of Oil Palm Fruit

The carotenoid content of the dried palm fruit ranged from 7.5 × 10^−5^ mg/g (solar-dried product) to 0.03 mg/g (oven-dried product) as represented in Table 3. Palm fruit has a rich orange-red colour due to its high content of carotene. The main carotenoids in palm oil fruit are *β*- and *α*-carotene, which are the precursors of vitamin A. However, low beta-carotene values could be influenced by the drying conditions. The oxidation of carotene leading to discolouration and bleaching is enhanced by hydroperoxides produced from lipid oxidation [4]. The extreme low values of solar-dried samples can be explained by *β*-carotene degradation, partly instigated by exposure to light or air. Crude palm oils in the palm fruit subjected to boiling had lower *β*-carotene contents [26]. However, low temperatures keep carotenoids protected from the elimination of oxygen and light [27].

The oven-dried samples recorded a higher antioxidant value of 61.80% with solar drying being the lowest, 21.46%. Antioxidants help food to maintain the levels of nutrients, texture, colour, taste, freshness and functionality, aroma, and appeal to consumers. However, drying causes loss of bioactive compounds and may pose an adverse effect on the nutrients, physical properties, and antioxidant activity of agricultural products [28].

## 4. Conclusion

The method of drying did influence the amount of crude fat in the oil palm fruit mesocarp but did not have much effect on the ash and crude protein because of less variation among the dried products. The dried mesocarp of the oil palm fruit may be stored for a longer period without spoilage through oxidative rancidity because of the low levels of % FFA and AV and high levels of antioxidants it possesses. Boiling the palm fruits at 100°C for 30 min before processing was responsible for essentially reducing the % FFA and AV in the palm fruit mesocarp. The overall drying method that presents the best option in terms of drying time and quality parameters was the electric oven. This study, therefore, provides useful information for the processing of palm fruit mesocarp in dry form to reduce the attack of fungi which affect the shelf life of the ripe palm fruits and further reduce the oil quality.

## Figures and Tables

**Table 1:** Proximate composition and metabolic energy of the oil palm fruit mesocarp at different drying treatments.

Sample	Moisture (%)	Ash (%)	Fat (%)	Protein (%)	CHO (%)	CME (kCal/100 g)
TTOD	1.49 ± 0.09^a^	0.69 ± 0.05^bc^	87.58 ± 0.53^g^	1.98 ± 0.31^b^	8.26 ± 0.70^b^	829.18 ± 0.43^g^
DTOD	2.35 ± 0.25^cd^	1.12 ± 0.03^e^	79.68 ± 0.19^d^	2.41 ± 0.26^bc^	14.45 ± 0.20^d^	784.56*d* ± 0.02^d^
TSD	1.64 ± 0.24^ab^	0.79 ± 0.05^cd^	90.03 ± 1.42^h^	2.03 ± 0.16^b^	5.51 ± 1.07^a^	836.16 ± 0.06^h^
DSD	2.13 ± 0.23^bc^	1.13 ± 0.07^e^	84.84 ± 0.12^f^	3.26 ± 0.27^e^	8.64 ± 0.45^b^	803.60 ± 0.04^f^
TOD	2.06 ± 0.33^abc^	0.76 ± 0.06^cd^	80.63 ± 0.16^de^	3.09 ± 0.26^de^	13.46 ± 0.81^d^	786.20 ± 0.05^de^
DOD	2.53 ± 0.20^cd^	1.15 ± 0.04^e^	82.03 ± 1.97^e^	3.40 ± 0.28^e^	10.90 ± 2.09^c^	795.47 ± 0.05^e^
TFD	2.88 ± 0.23^de^	0.53 ± 0.05^a^	78.10 ± 0.68^c^	3.37 ± 0.25^e^	15.12 ± 0.15^de^	775.96 ± 0.04^c^
DFD	3.28 ± 0.18^e^	0.78 ± 0.02^cd^	90.60 ± 1.66^h^	0.93 ± 0.26^a^	4.41 ± 1.55^a^	836.76 ± 0.03^h^
TC	26.36 ± 0.48^f^	0.60 ± 0.02^ab^	53.81 ± 0.62^b^	2.31 ± 0.25^bc^	16.92 ± 0.10^e^	561.21 ± 0.04^b^
DC	31.15 ± 0.31^g^	0.81 ± 0.06^d^	49.79 ± 0.48^a^	2.66 ± 0.29^cd^	15.59 ± 0.17^de^	521.11 ± 0.05^a^

Values represent mean ± SD of at least three replicates. Means in the same column with the same superscript are not significantly different (*p* > 0.05). CHO: available carbohydrate; CME: calculated metabolic energy; TTOD: tenera traditional oven dried; DTOD: dura traditional oven dried; TSD: tenera solar dried; DSD: dura solar dried; TOD: tenera oven dried; DOD: dura oven dried; TFD: tenera freeze dried; DFD: dura freeze dried; TC: tenera control; DC: dura control.

**Table 2:** Effect of different drying methods on the physicochemical properties of oil palm fruit mesocarp.

Sample	Acid value (mgKOH/g)	Free fatty acid (%)	Titratable acidity (%)	Peroxide value (meq/kg)	pH @25.60°C
TTOD	3.00 ± 0.10^b^	1.5 ± 0.11^b^	0.35 ± 0.00^a^	3.28 ± 0.21^bc^	5.13
DTOD	3.92 ± 0.13^cd^	1.97 ± 0.12^cd^	1.06 ± 0.03^d^	3.35 ± 0.28^c^	5.28
TSD	7.05 ± 0.19^e^	3.55 ± 0.20^e^	0.53 ± 0.01^b^	17.81 ± 0.98^e^	4.48
DSD	8.83 ± 0.39^f^	4.44 ± 0.03^f^	1.07 ± 0.02^d^	7.08 ± 0.03^d^	4.61
TOD	2.53 ± 0.03^ab^	1.27 ± 0.02^ab^	0.53 ± 0.01^b^	3.37 ± 0.86^c^	5.56
DOD	4.12 ± 0.07^d^	2.07 ± 0.06^d^	0.35 ± 0.01^a^	2.08 ± 0.14^a^	5.57
TFD	2.17 ± 0.05^a^	1.09 ± 0.04^a^	1.06 ± 0.01^d^	1.92 ± 0.02^a^	5.73
DFD	3.52 ± 0.36^c^	1.77 ± 0.04^c^	0.71 ± 0.01^c^	2.48 ± 0.13^abc^	5.24
TC	2.20 ± 0.03^a^	1.11 ± 0.02^a^	0.54 ± 0.00^b^	2.25 ± 0.45^ab^	5.48
DC	4.17 ± 0.42^d^	2.10 ± 0.30^cd^	0.52 ± 0.00^b^	3.35 ± 0.51^c^	5.2

Values represent mean ± SD of at least three replicates. Means in the same column with the same superscript are not significantly different (*p* > 0.05). TTOD: tenera traditional oven dried; DTOD: dura traditional oven dried; TSD: tenera solar dried; DSD: dura solar dried; TOD: tenera oven dried; DOD: dura oven dried; TFD: tenera freeze dried; DFD: dura freeze dried; TC: tenera control; DC: dura control.

**Table 3:** Beta-carotene and antioxidant properties of oil palm fruit mesocarp.

Samples	Beta carotenoid (mg/g)	Antioxidant (%)
TTOD	0.03 ± 0.00^de^	37.15 ± 0.05^a^
DTOD	0.03 ± 0.00^f^	46.08 ± 0.06^e^
TSD	4.3 × 10^−4^ ± 0.00^a^	21.46 ± 0.02^b^
DSD	7.5 × 10^−5^ ± 0.00^a^	43.90 ± 0.05^d^
TOD	0.03 ± 0.00^ef^	48.61 ± 0.00^c^
DOD	0.02 ± 0.00^bc^	61.80 ± 0.03^f^
TFD	0.02 ± 0.00^bcd^	38.47 ± 0.19^g^
DFD	0.02 ± 0.00^b^	52.59 ± 0.06^h^
TC	0.02 ± 0.01^cd^	27.13 ± 0.03^i^
DC	0.03 ± 0.00^d^	38.23 ± 0.22^j^

Values represent mean ± SD of at least three replicates. Means in the same column with the same superscript are not significantly different (*p* > 0.05). TTOD: tenera traditional oven dried; DTOD: dura traditional oven dried; TSD: tenera solar dried; DSD: dura solar dried; TOD: tenera oven dried; DOD: dura oven dried; TFD: tenera freeze dried; DFD: dura freeze dried; TC: tenera control; DC: dura control.

## Data Availability

All data are included in the manuscript.
